# Analysis of DUX4 Expression in Bone Marrow and Re-Discussion of DUX4 Function in the Health and Disease

**DOI:** 10.5146/tjpath.2021.01564

**Published:** 2022-09-15

**Authors:** Ceren Hangul, Oznur Tokta, Sibel Berker Karauzum, Bahar Akkaya, Hulya Yıldırım, Funda Tayfun Kupesiz, Ayse Nur Akınel

**Affiliations:** Department of Medical Biology and Genetics, Akdeniz University, Faculty of Medicine, Antalya, Turkey; Department of Pathology, Akdeniz University, Faculty of Medicine, Antalya, Turkey; Department of Pediatric Hematology and Oncology, Akdeniz University, Faculty of Medicine, Antalya, Turkey

**Keywords:** DUX4, Bone marrow, Hematopoietic progenitor cells, B-ALL, Cancer, Facioscapulohumeral muscular dystrophy

## Abstract

***Objective:*** DUX4 is an embryonic transcription factor (TF) later silenced in somatic tissues, while active in germline testis cells. Re-expression in somatic cells has been revealed to be present in pathologic conditions such as dystrophy, leukemia, and other cancer types.

Embryonic cells, cancer cells and testis cells that show DUX4 expression are pluri-multipotent cells. This lead us to question “Could DUX4 be a TF that is active in certain types of potent somatic cells?” As a perfect reflection of the potent cell pool, we aimed to reveal DUX4 expression in the bone marrow.

***Material and Method:*** Bone marrow aspiration materials of seven healthy donors aged between 3 and 32 (2 males/5 females) were investigated with qPCR analysis after RNA isolation for the presence of *DUX4* full length mRNA expression. Samples have been investigated for protein existence of DUX4 via immunohistochemistry in two donors that had sufficient aspiration material.

***Results:*** *DUX4* mRNA expression was present in all donors, with higher expression compared to *B-actin*. DUX4 positive stained cells were also detected by immunohistochemistry.

***Conclusion:*** With these results, novel expression for DUX4 in hematopoietic tissue is described. Further studies on the function of DUX4 in hematopoietic cells can shed light on DUX4-related pathways, and contribute to the treatment of DUX4-related diseases such as B-ALL, other cancers, and facioscapulohumeral muscular dystrophy.

## INTRODUCTION

DUX4 is a double homeobox transcription factor (TF) that is active in the embryonic period (1–3). It is located on D4Z4 repeat units on chromosome 4q35 (4). In the normal condition, D4Z4 repeat ranges from 11 to 100 units and each of these units consists of 3300 bases (5). At the end of each 3300 bases, *DUX4* gene sequence is settled (4). From this sequence, two main isoforms, a long isoform called DUX4-fl (full length) and a short isoform called DUX4s are transcribed with alternative splicing. Expression profiles and roles of these isoforms are divergent. DUX4s can be expressed in some of the somatic cells; however DUX4-fl is expressed only in embryonic cells and germline cells of healthy adults (6,7). Expression of DUX4-fl in somatic tissue is observed in facioscapulohumeral muscular dystrophy (FSHD) and has been revealed to be pathogenic (7). In FSHD, there is a contraction of D4Z4 repeats on chromosome 4 (8). Contraction leads to re-activation of the *DUX4* gene in somatic skeletal tissue (9). When this contraction is accompanied by the qA allele in the same chromosome (10), re-activated *DUX4* mRNA is stabilised, which is toxic to the skeletal cell (9) leading to apoptosis (11). Other than FSHD, few studies have revealed rare expression of DUX4-fl in somatic cells such as keratinocytes and thymic cells (12,13). Recent searches on cancer have also revealed that DUX4 expression was present in most of the cancer cell types suggesting new roles for DUX4. First studies arrived with the report on *CIC-DUX4* fusion in sarcomas (14). Later on, Chew et al. revealed that DUX4-fl is actively expressed in 25 different cancer types (15). B-ALL is one of the other newly discovered DUX4-expressing cancer types (16). In ALL, the most common genetic DUX4 related event is *DUX4* fusion with other ALL specific genes such as *ERG* (17). This fusion mostly causes DUX4 overexpression leading to cell transformation and B-ALL (18). However, detailed DUX4 related pathophysiologic mechanisms in B-ALL are not known yet (16). Interestingly, there is no information on whether DUX4 expression is present in healthy hematopoietic tissue. For this reason, in this study we investigated the presence of DUX4-fl expression in bone marrow aspirates of healthy donors. By investigating multipotent healthy hematopoietic sample, we aimed to get an answer for two main questions: i) Like its expression in malignant hematopoietic cells, is DUX4 expression present in healthy hematopoietic cells? and ii) Is DUX4-fl expression present in somatic undifferentiated cell types with high proliferation potential?

## MATERIAL and METHOD

This study had been approved by the Akdeniz University Faculty of Medicine Clinical Research Ethical Committee; Decision Number: 1087, Date: 27.11.2019

### Donors

Two males, five females, a total of seven healthy bone marrow donors have been included in this study. Their ages varied between 3 and 32. Age and gender information of the donors have been summarised in Table 1.

**Table 1:** Gender and age of healthy donors.

**Donors**	**Age**	**Gender**
Donor 1	3	female
Donor 2	4	female
Donor 3	9	female
Donor 4	10	male
Donor 5	16	female
Donor 6	23	male
Donor 7	32	female

### Bone Marrow Sampling

After the application of topical anaesthesia, bone marrow material has been obtained with superior iliac crest aspiration.

### RNA extraction

Total RNA isolation from bone marrow samples has been performed with the Qiamp RNA blood mini kit (Qiagen).

### cDNA synthesis

Obtained total RNA was converted into cDNA using a Blue-Ray PCR device in accordance with the protocol with Applied Biosystems High-Capacity cDNA Reverse Transcription Kit (4368814). The converted cDNA samples were stored at -20°C. Reverse transcriptase PCR conditions were: 25oC 10 minutes, 37oC 120 minutes, 85oC 5 minutes, 4oC ∞.

### Spectrophotometric Measurement of cDNA Samples

Amount and purity measurements of the isolated cDNA samples were determined with a spectrophotometer (Quawell / Q9000B). For the measurement, 2 μl of cDNA sample was loaded into the spectrophotometer device. The purity and amount of cDNA were determined in ng/μl with the ratio of measurements obtained at 260 nm and 280 nm wavelengths of the samples. cDNA samples were diluted to 100 ng/μl for use in PCR studies.

### Quantitative Real-Time Polymerase Chain Reaction (qRT-PCR)

Applied BiosystemsTM StepOnePlusTM Real-Time PCR device and PowerUpTM SYBRTM Green Master Mix (A25776) kit were used in accordance with the protocol to determine the level of *DUX4* gene expression. Mixes containing the cDNAs of each donor were added to the 96-well plate in at least three replicates. For the analysed *DUX4* gene, a negative control plate containing no cDNA was loaded. The *B-actin* gene, which is the housekeeping gene, was used as control gene in the analyses. The primer pair for *B-actin* is 5’-CCTGGCACCCAGCACAAT-3’(forward) and 5’-GCCGATCCACACGGAGTACT-3’ (reverse). The primer sequences used for the *DUX4-fl* are CAAGGGGTGCTTGCGCCACCCACGT (forward) and GGGGTGCGCACTGCGCGCAGGT (reverse). qPCR conditions were: 95oC 10 minutes, 95oC 15 seconds, 60oC 1 minute, 40 cycles.

qPCR analysis had been performed at least three times for each sample. The average of the results was calculated and integrated into the graphic by converting into the nearest integer.

### Agarose Gel Electrophoresis

To control the presence of *DUX4* cDNA conversion and amplicon size of *DUX4-fl *transcript, RT-PCR products were run on 2% agarose gel.

### Immunohistochemistry

Bone marrow aspiration materials from two donors were immunohistochemically stained for DUX4 using rabbit monoclonal IgG E5-5 antibody (cloneP4H2) raised against a synthetic peptide corresponding to the C-terminus of the human DUX4 protein (catalog no ab124699; Abcam). E5-5 antibody recognizes the C terminal domain of DUX4 (19). An automated DAKO Omnis staining platform was used to perform all immunohistochemical procedures, with the aid of the Optiview detection kit. Antibody staining was performed at 1:50 dilution.

## RESULTS

### All of Seven Donors had *DUX4* mRNA Expression

**Figure 1:**
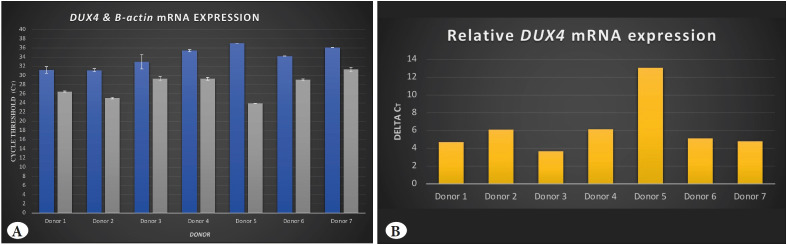
**A)** qPCR analysis of seven healthy donors with different ages and gender. Blue bars represent DUX4 mRNA expression and grey bars represent housekeeping B-actin mRNA expression. **B)** DeltaCT values of DUX-fl mRNA expression in bone marrow aspirates.

mRNA expression of *DUX4* was revealed to be present in each of the bone marrow samples. *DUX4* expression level exhibited similar levels in between the bone marrow samples of donors and no significant difference was noted depending on age and gender (Figure 1). In addition, the expression level of *DUX4* was higher in each sample compared to the level of *B-actin* expression (Figure 1).

### *DUX4* Protein Staining was Positive in the Cells that were Relatively Larger

**Figure 2:**
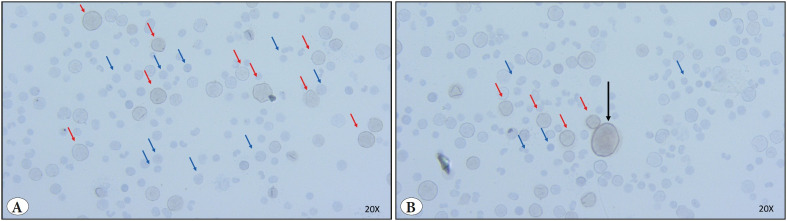
DUX4 Immunohistochemistry figures of bone marrow aspirates of Donor 2. **A)** Red arrows indicate brown cells that are positive for DUX4 protein, blue arrows indicate DUX4 negative cells. **B)** Red arrows indicate DUX4 positive cells, blue arrows indicate DUX4 negative cells, black arrow indicates DUX4 positive cell with its huge nucleus. All photographs have been taken 20X microscopic magnification.

**Figure 3:**
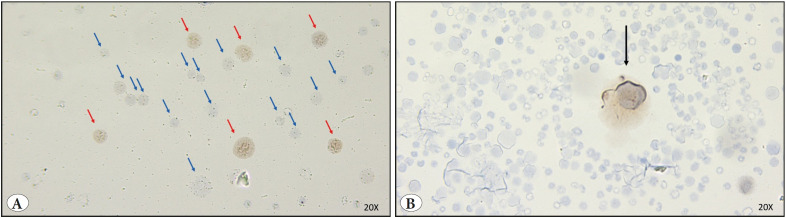
DUX4 Immunohistochemistry figures of bone marrow aspirates of Donor 3. **A)** Red arrows indicate brown cells that are positive for DUX4 protein, blue arrows indicate DUX4 negative cells. **B)** Black arrow indicates DUX4 positive huge cell with an empty cytoplasmic area around, indicating a megakaryocyte. All photographs have been taken 20X microscopic magnification.

Donor 2 and donor 3, who had sufficient aspiration material, were also investigated with DUX4 protein staining. Both donor 2 and 3 had a portion of positively staining cells in their bone marrow aspirates. Staining density was low. Positively stained hematopoietic cells were observed to be larger in size relative to negatively stained cells in bone marrow aspirates (Figure 2 Figure 3).

Positive staining of two huge cells revealed a positive expression of DUX4 protein in megakaryocytes (Figure 3).

## DISCUSSION

DUX4-fl is an active TF expressed in embryonic pluripotent cells (2,3,20). Normal function of DUX4-fl is attributed to early developmental stages. However recent proofs of DUX4-fl expression in late-differentiating keratinocytes (12), cells in the thymus (13), and lymphoblastoid cells (21,22) have revealed that DUX4-fl expression is present in somatic and in differentiating cells. Human mesenchymal stromal cells (hMSC) (23) and mesangioblasts of facioscapulohumeral muscular dystrophy (FSHD) (24) are the other somatic/potent cells revealed to express DUX4. As another supporting data, some types of cancer cells, which are mostly somatic and have a high proliferation rate, were positive for DUX4 and the first study came with *CIC*/*DUX4* fusion (14). In another comprehensive study, Chew et al. revealed DUX4-fl was expressed in 25 different types of cancer cells (15). Interestingly, B-ALL was one of the DUX4 related cancers (17,25–27). It was shown that DUX4 expression lead defective B cell differentiation and transformation (18,28). All aforementioned findings lead us to investigate DUX4-fl expression in healthy multipotent/progenitor hematopoietic cells, which has not been revealed before.

DUX4 is a pioneer transcription factor and has essential roles in zygotic gene activation (1–3). Pioneer transcription factors initiate cell differentiation, and they activate cell specific genes. Pioneer TFs need to be expressed at certain time points and in precise amounts: they have spatiotemporal speciality. For example, PAX7 is another homeobox TF (29) that has exchangeable domains with DUX4 (30). Mayran et al. have revealed that even transient PAX7 expression is sufficient for the cell fate (29). Similar to that, the quantity of expression level (31) and spatiotemporal specificity (32) are critical for DUX4 expression. Expression of transient bursts of stochastic expression in a small proportion of FSHD myonuclei (6) especially supports the spatiotemporal pattern of DUX4.

DUX4 binds to its target genes via its N terminal domain (4) and activates transcription via its C terminal domain by attracting histone acetyl transferase (HAT) complexes to that area (33). DUX4s does not contain a C terminal while DUX4-fl contains a C terminal domain and can activate gene expression. In addition, DUX4s is normally expressed in somatic tissues, while DUX4-fl is assumed not to be expressed in healthy somatic tissues (6). Because of these, we preferred to investigate DUX-fl in somatic hematopoietic cells, and used *DUX4-fl* specific primers for mRNA analysis and antibody that recognises C-terminal domain for the protein analysis. Two males and five females aged between 3 to 32 were analysed for DUX4 expression. As a result, in mRNA level, we detected that *DUX4-fl* were present and expression levels were close to each other in all of the samples without any exception. Remarkably, expression of *DUX4-fl* mRNA levels was higher compared to *B-actin* levels (Figure 1). No significant difference was present in the expression of DUX4 depending on age and gender factors; except a relative higher level in Donor 5 who was an adolescent female. This may be related to effect of estradiol on DUX4 that had been revealed in FSHD studies in skeletal tissue (34–36). It can be interesting to investigate this in detail in future studies. Regardless of non-significant level differences, it might be said that our data revealed present *DUX4-fl* expression in seven healthy somatic bone marrow cells without exception, independent of age and gender factors. This novel data can indicate that *DUX4-fl* has evident function(s) in somatic healthy hematopoietic cells.

In order to observe whether DUX4 expression is present at the protein level in the hematopoietic cells, we performed immunohistochemical staining of the semi-liquid aspirates of two donors that had remaining aspiration materials. In these preparations, slight staining has been observed (Figure 2 Figure 3), indicating a low expression level of DUX4-fl similar to that observed in FSHD cells (6,9,37). Slight staining is compatible with its pioneer role in other cell types. This slight positive staining was detected in not all but some part of the cells (Figure 2 Figure 3) and was remarkably larger in size compared to negatively stained ones (Figure 2 Figure 3). In hematopoietic tissue, larger cells indicate progenitor cells that are in the earlier stages of differentiation (38). Because of that, positive staining in the cells with larger nuclei might suggest that DUX4 is an active TF especially in progenitor hematopoietic cells that came into play in earlier stages and that might be in the less differentiated status. Partial positive staining of the cell population might also support the spatiotemporal expression of DUX4. Interestingly, the observed megakaryocytic cells that had staining with a huge nucleus (Figure 3) may indicate an additional role of DUX4 for thrombocyte function. DUX4 expression in megakaryocytic cells has not been revealed before and might be valuable for understanding thrombocyte-related diseases. Because of this, it is worthwhile and necessary to identify cell type specific expression of DUX4 in future studies.

Since its direct genetic relationship and numerous related studies in literature, FSHD provides most of the information on the function of DUX4. In a developmental model on FSHD it was suggested that DUX4-fl is normally expressed in early development and suppressed during cellular differentiation (6). However, recent results indicate that DUX4-fl is also expressed in later phases of cellular differentiation. Gannon et al. have revealed that late-differentiating keratinocytes expressed DUX4 (12). Jones and Banerji et al. have revealed DUX-fl expression in lymphoblastoid cells (21,22) and Das and Chadwick have revealed DUX4 expression in the cells of thymus (13). It was revealed that some of the healthy muscle- derived cells also exhibited DUX4 expression (31). These cells might be in the later phases of differentiation. Supporting that, we observed easier detection of DUX4 protein in first passages (observational data) in our previous study on *in vitro *estradiol treatment in FSHD cell culture (36). On the other hand, with the presence of DUX4-fl in pluripotent hematopoietic cells, this present study revealed that expression of DUX4-fl not only specific to late differentiating somatic cells, but it can also be expressed in the earlier phases of cellular differentiation. Supporting this, DUX4 expression in early differentiation was revealed to be present also in hMSCs (23). Expression in the earlier phases of differentiation might shed light on other DUX4 related diseases such as FSHD. Deficiency or spatiotemporal disturbance of DUX4 at earlier stages of cell differentiation might explain FSHD. Supporting that DUX4-fl expression in differentiated skeletal cells is not sufficient for FSHD to occur (31). Our results might signify a hypothesis: deficiency or disturbance in the critical spatiotemporal timing and amount-quantity of DUX4 expression could result in pathology in the precursor potent cells at earlier stages and end up with a decreased healthy cell pool. Observation of molecular disease markers in fetal FSHD muscles is compatible with early disturbance of DUX4 (39). Stabilization or de-repression of re-expressed DUX4 via accomplishing qA allele, *SMCHD1 *or* DNMT3B *or* LRIF1* mutations (40–42) can contribute to spatiotemporal disturbance of DUX4 and lead to the DUX4 toxicity observed in later phases of differentiation. There are multiple treatment trials that aim to inhibit DUX4. Inhibition of DUX4 can prevent these cells from going into apoptosis by eliminating DUX4 toxicity in differentiated FSHD cells. However, it might not provide sufficient clinical improvement in case of a deficiency or disturbance of DUX4 in the early stages. Additionally and importantly, DUX4 inhibition might cause side effects related to the aforementioned cell types that actively need DUX4 expression.

**Figure 4:**
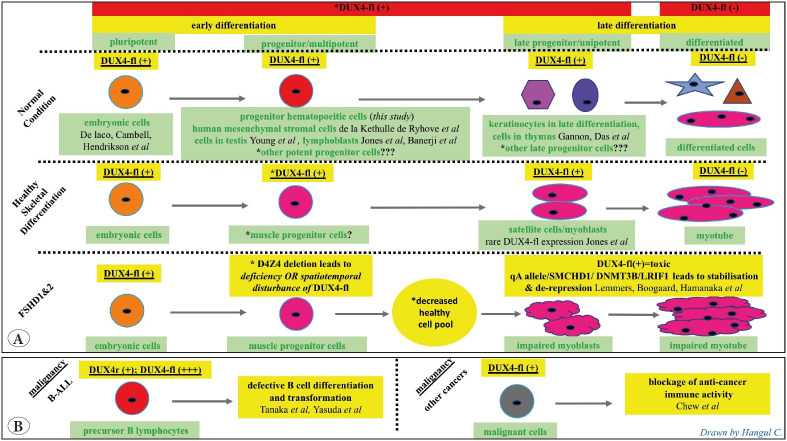
Schematic presentation for the DUX4 role in **(A)** healthy hematopoietic cells, healthy embryonic cells, healthy skeletal differentiation and FSHD; **(B)** in malignancy. Revealed knowledge referred with authors.

In summary, with the present study it was shown that DUX4 is an active TF in progenitor hematopoietic cells. We suggest that DUX4 expression in earlier stages of cell differentiation can be critical, and DUX4 deficiency and/or spatiotemporal DUX4 disturbance might be related to the pathology of diseases (Figure 4).

In conclusion, DUX4-fl, which is assumed to have rare expression in somatic cells, was found to be present in healthy bone marrow aspirates at both the mRNA and protein level in this study. With this data, it was revealed that the DUX4 expression shown in hematologic malignancy, which is said to be re-expressed in B-ALL, is already expressed by some part of healthy bone marrow cells. The obtained data from this study indicate that the expression of DUX4 should be reviewed and studied in all tissues in future studies, especially in progenitor potent cells. Clarifying the role of DUX4 in potent healthy somatic cells might provide key information for the pathophysiology of B-ALL, other DUX4 related cancers, and FSHD. With further information, more comprehensive treatment strategies can be developed in DUX4 related diseases.

## Study Limitations

Since it is an invasive approach, bone marrow sampling in a separate study is not ethical. Therefore, this study had been carried out using bone marrow samples from healthy bone marrow donors, remaining after transplantation. Because of this, a limited amount and number of materials could be examined.

## Conflict of Interest

All authors declare that they have no conflict of interest.
